# The Link Between Structural and Functional Brain Abnormalities in Depression: A Systematic Review of Multimodal Neuroimaging Studies

**DOI:** 10.3389/fpsyt.2020.00485

**Published:** 2020-06-03

**Authors:** Dominique S. Scheepens, Jeroen A. van Waarde, Anja Lok, Glenn de Vries, Damiaan A. J. P. Denys, Guido A. van Wingen

**Affiliations:** ^1^Department of Psychiatry, Amsterdam Neuroscience, Amsterdam UMC, University of Amsterdam, Amsterdam, Netherlands; ^2^Department of Psychiatry, Rijnstate Hospital, Arnhem, Netherlands; ^3^Amsterdam Brain and Cognition, University of Amsterdam, Amsterdam, Netherlands

**Keywords:** major depression, structural and functional abnormalities, neuroimaging, systematic review, relation structure and function in MDD

## Abstract

**Background:**

Adequate and timely identification of depression is essential to improve patient care. A potential method to achieve this is by using neuroimaging. Many neuroimaging studies have revealed widespread abnormalities in brain structure and function in patients with depression, but in most studies only single neuroimaging modalities were used. Links between abnormalities in brain structure and function need to be therefore further explored in order to define diagnostic and therapeutic applications.

**Methods:**

A systematic literature review according to preferred reporting items for systematic reviews and meta-analyses (PRISMA) guidelines was conducted.

**Results:**

Out of 2,516 articles, only 14 studies were eligible to be included. These studies combined structural and functional neuroimaging methods in depressed patients compared to controls. Four studies reported a negative relationship between brain structure and function within the default mode network: reduced gray or white matter integrity in depressed patients compared to healthy controls was associated with enhanced neural activity or connectivity. The other studies reported positive relationships (two studies), mixed relationships (two studies), or no link (six studies) between structural and functional brain abnormalities.

**Conclusion:**

This systematic literature review revealed no robust relationship between abnormalities in brain structure and function in patients with depression. Remarkably, only 14 studies could be included and four of these suggested enhanced default mode network connectivity associated with reduced structural brain integrity. In the ongoing development of the diagnostic and treatment applications of neuroimaging, large-scale studies that combine structural with functional neuroimaging are required to determine the relationship between structural and functional abnormalities in depression.

## Introduction

Depression is the second leading cause of disability worldwide and one of the most prevalent psychiatric disorders (1). Clinical heterogeneity is an important characteristic of this condition. Adequate and timely establishment of depression is essential, because shorter duration of untreated depression is associated with favorable treatment outcomes in patients (2). A broader understanding of underlying pathophysiology of depression is therefore fundamental to detect (and hopefully to prevent) this condition in an early stage and to further distinguish phenotypes within this heterogeneous clinical syndrome. Various neuroimaging methods have been used to explore distinct underlying neural mechanisms of depression. These have been based on several proposed neurobiological models and are mostly aimed to localize specific structural and functional abnormalities. Although the use of modern neuroimaging techniques seems promising, no conclusive diagnostic nor treatment outcome predictive applications are implemented in clinical practice yet.

**Figure 1:**
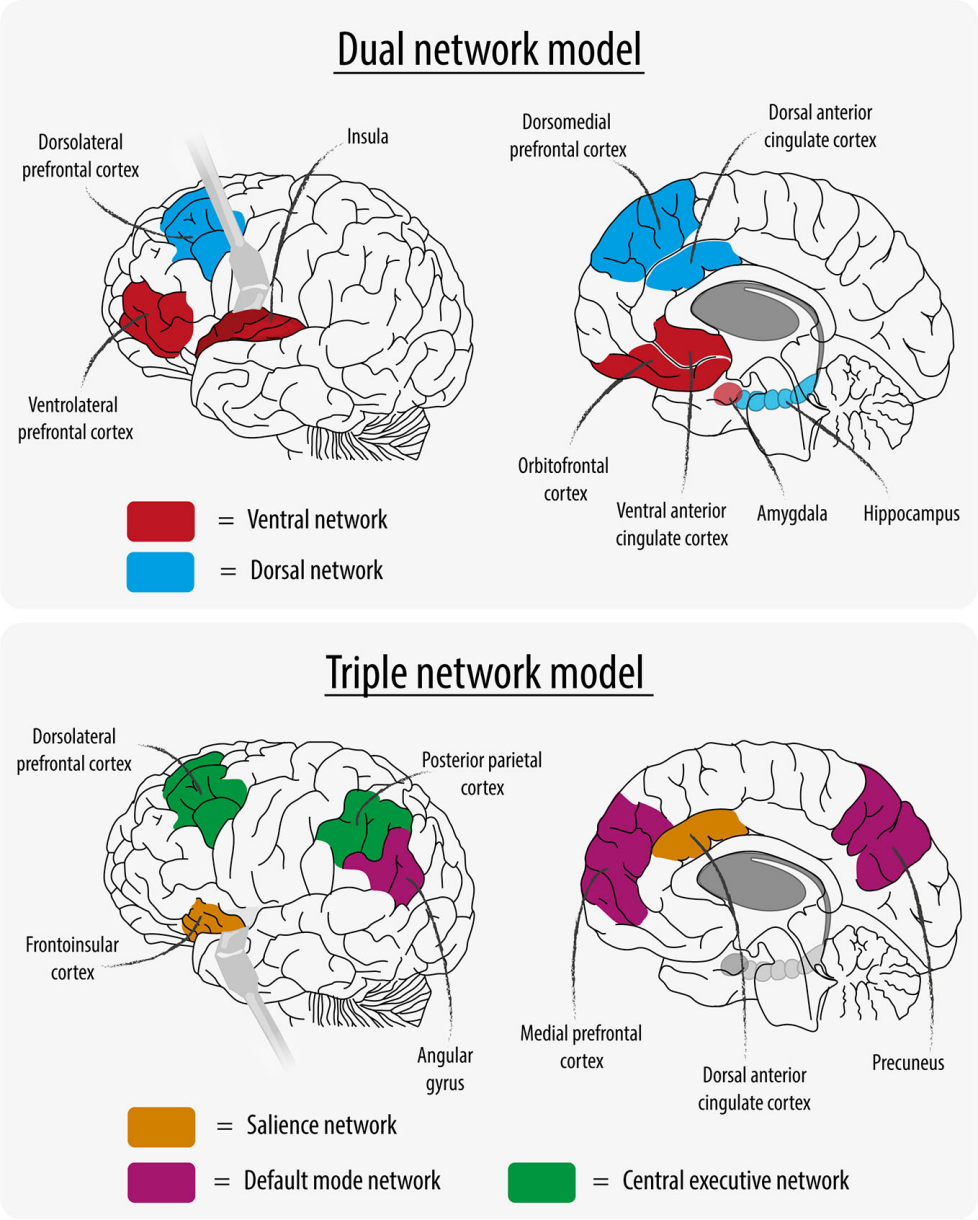
Network Models.

More than 20 years ago, Mayberg (3), and later Philips et al. (4), proposed the “dual network model” of depression consisting of a ventral and a dorsal network (**Figure 1**). The ventral network was hypothesized to mediate vegetative and somatic symptoms and consisted of hyperactive brain regions, whereas the dorsal network was thought to mediate cognitive aspects of depression and consisted of hypoactive brain regions. More recently, data-driven extraction of maximally independent neural networks using independent component analysis (ICA) identified highly reproducible intrinsic connectivity networks (5, 6). Menon thus (7) proposed the “triple network model” for depression, in which three functional brain networks are described: the “central executive network” (CEN) involved in higher-order cognitive processes, the “default mode network” (DMN) which is primarily active at rest, and the “salience network” (SN) which is important for shifting attention to relevant stimuli (see **Figure 1**). With this paradigm, the focus in recent basic research shifted from the hypothesis that depression is a dysregulation between dorsal and ventral networks to the concept of dysregulation between and within large-scale brain networks. This shift created a new chance in developing clinically useful diagnostic and/or predictive neuroimaging applications for depression.

Clusters of depressive symptoms are nowadays increasingly understood as multiple brain network disorders with widespread alterations in brain structure and function (8, 9). “Rumination,” for example, is associated with dysfunction in parietal components of the DMN, “emotional disinhibition” with dysfunction in the CEN, and “emotional over-reactivity” with dysfunction of the SN (2). Compared to healthy controls, meta-analyses of studies in depressed patients reveal consistent abnormalities in gray matter volume (10–14), white matter tracts brain responses to emotional stimuli (15, 16), functional brain responses to cognitive stimuli (17), and in functional connectivity across distant brain regions (18, 19). It still however, remains unclear if and how these structural and functional brain abnormalities in depressed patients are related. More insight into these relationships may improve (earlier) detection while also providing a clearer organization and definition of the different phenotypes of depression in patients.

In studies of healthy individuals, a strong positive relationship has been found between regions with evident structural brain connections and resting state functional connectivity (20). Furthermore, integrity of white matter fibers connecting the amygdala to the prefrontal cortex is found to be related to individual differences in amygdala responsivity (21, 22). In depressed patients however, brain volume and structural connectivity are typically found to be reduced (9, 10). Moreover, differences in neural activity and functional connectivity vary across different depression-related brain networks (17, 23). Until now several neuroimaging techniques have been used to examine functional abnormalities in depressed patients *versus* controls. This will be discussed below in relation to the neurobiological models of depression.

Meta-analysis of resting-state blood flow and metabolism studies in depressed patients *versus* controls show on the one hand decreased activation of the anterior cingulate cortex (ACC), posterior cingulate cortex (PCC), middle frontal gyri, insula, and left superior temporal gyrus, and on the other hand increased activation of the thalamus, caudate nucleus, and medial, inferior, middle, and superior frontal cortex (24). More recently, a meta-analysis reported only hyperactivity of the thalamus in depressed patients. Two studies demonstrated hyperactivity in the subcallosal gyrus, right dorsolateral prefrontal cortex (PFC), and parahippocampal gyrus, and hypoactivity in the left dorsolateral PFC and dorsal ACC (15). As predicted by the “dual network model” (18, 19), both hypo- and hyperactivity in distinct brain areas were reported.

Several studies have focused on functional connectivity between and within large-scale brain networks. A meta-analysis of resting-state functional magnetic resonance imaging (RS-fMRI) studies reported on the one hand hypoconnectivity within the CEN and between the SN and the precuneus, and on the other hand hyperconnectivity within the DMN and between the DMN and dorsolateral PFC (18). In line with the “triple network model” (7), these results indicate that depression is characterized by dysregulation of large-scale brain networks. Furthermore, these results suggest that activity is increased within networks that are important for directing attention to the internal world (DMN), whereas activity is reduced within brain networks that are important for directing attention to the outside world (SN) and performing goal-directed actions (CEN).

These meta-analyses of unimodal neuroimaging studies suggest that there are consistent differences in brain structure and function in patients with depression compared to healthy controls. It still remains however, unclear whether and how these abnormalities are related. In this systematic review we therefore report the results from multimodal neuroimaging studies on structural and functional brain abnormalities in depressed patients *versus* healthy controls. In doing so we hope to increase our understanding of the underlying pathophysiology which ultimately might lead to benefits for clinical use.

## Methods

### Literature Search

**Figure 2:**
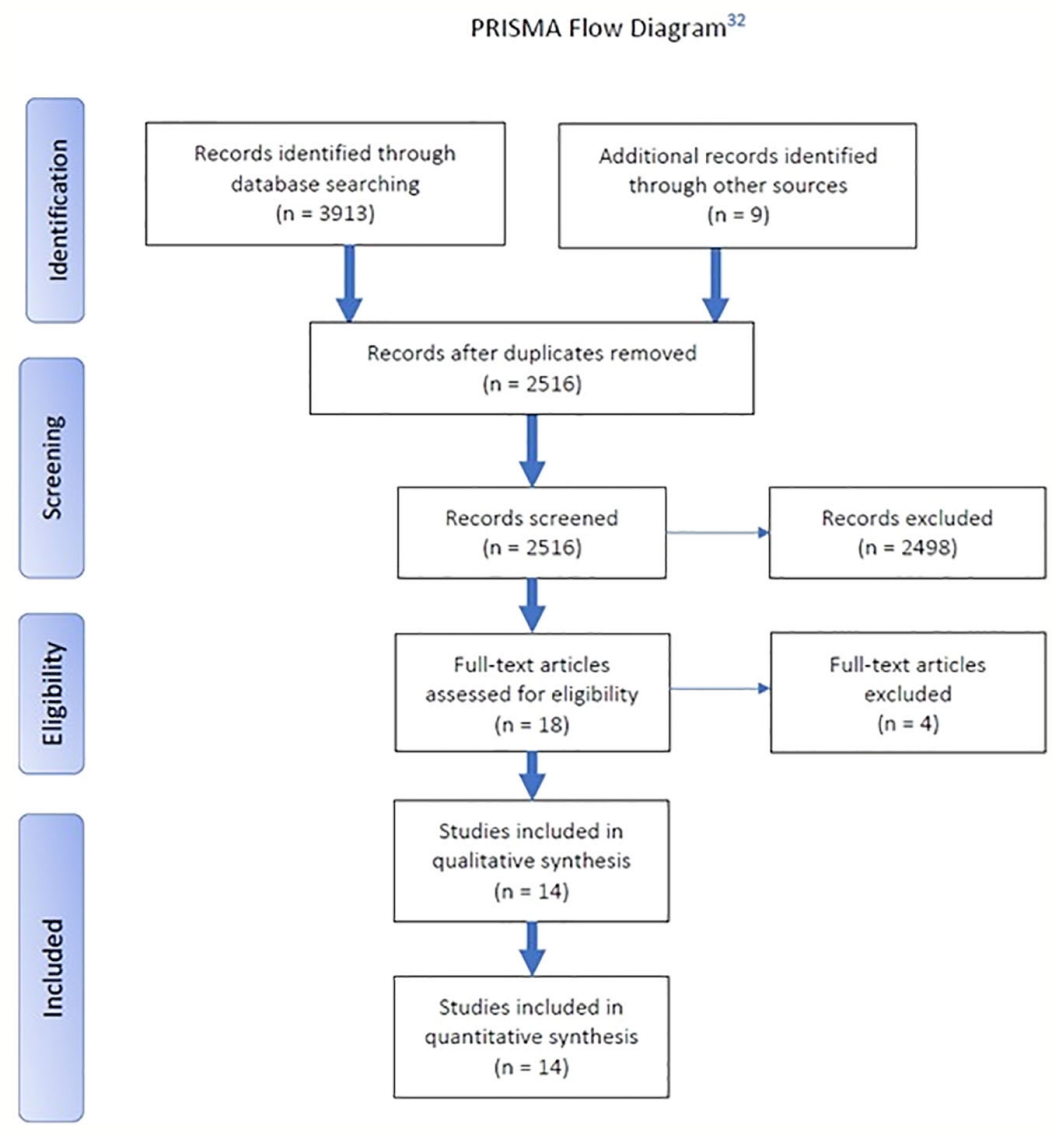
Preferred reporting items for systematic reviews and meta-analyses (PRISMA) flow diagram.

A systematic electronic literature search according to preferred reporting items for systematic reviews and meta-analyses (PRISMA) guidelines was performed (**Figure 2**). Databases Embase, MEDLINE, and PsycINFO were consulted on four separate occasions, and a final search was conducted on February 2, 2019. Major depressive disorder (MDD) synonyms or closely related topics were consulted: “MDD,” “major depressive,” “major depression,” “depressive” or “depression” (in conjunction with “disorder”), “treatment-resistant” or “recurrent” or “reactive” (combined with “depression”), “unipolar” and “bipolar.” This was narrowed down by using synonyms for “magnetic resonance spectroscopy,” “diffusion tensor imaging,” “arterial spin labeling,” “blood oxygen level-dependent,” “functional imaging,” and “functional connectivity,” “structural imaging,” and “structure” (individually or in conjunction with “abnormalities”). These synonyms were adapted into phrases and keywords, best suited for the corresponding database (see **Supplementary Tables 1–3**).

### Screening and Selection

The systematic search retrieved 2,516 articles, which were independently assessed in Rayyan QCRI by two authors (DS and GdV) and screened by title and/or abstract. Articles were eligible for inclusion if they met all the following criteria: research on 1) functional and 2) structural abnormalities (i.e., each deviation from what is normal or usual in the control group) in 3) MDD patients [unipolar (UD) and bipolar (BD) depressed patients] using 4) fMRI and 5) structural MRI. Exclusion criteria were: animal studies, case studies and studies with patients under the age of 18 years, and studies lacking a control group. After independent screening we compared and discussed our results during a follow-up meeting and resolved remaining conflicts. In total, 2,498 articles had to be excluded, mostly because of the lack of a control group and/or lack of analyses of the relationship between structural and functional neuroimaging. Next, 18 articles were thoroughly read in full text by both researchers for a final and more precise screening and inclusion. One article had to be excluded for using infant/adolescent patients, two for irrelevance concerning the research topic, and because it did not include a control group. After screening and selection, 14 articles were eligible to be included in the systematic review.

### Statistical Analysis

To examine the heterogeneity of included studies, several descriptive variables of the included studies were analyzed *post-hoc* (year of publication, total N, use of task-based fMRI, use of model-driven region-of-interest analyses, and the origin of the study). Descriptive statistics (χ^2^-tests and Mann-Whitney tests) were used to compare studies that found a relationship (negative, positive, or mixed) *versus* those that did not.

## Results

The fourteen included studies were published between 2011 and 2018. Sample sizes ranged from 17 to 461 participants per group (median = 36). The studies were conducted in Europe (N = 6), Asia (N = 5), or North-America (N = 3). Most studies (64%) used voxel-based analysis of gray matter (voxel-based morphometry; VBM) as structural measure, and fMRI as functional measure (93%) (**Supplementary Table 4**). Six of the 14 studies included both uni- and bipolar depressed patients. The largest sample size included consisted of only unipolar depressed patients (25) (see **Supplementary Table 5**). Studies used the Structured Clinical Interview for Diagnostic and Statistical Manual of Mental Disorders (DSM)-IV (26), Hamilton Ratings Scale for Depression (HDRS) (27) and Beck Depression Inventory-II (BDI-II) (28) to diagnose MDD. The included studies could be grouped by the relationship that was observed between structural and functional abnormalities. This relationship was most often assessed by visualizing the overlap between results for each modality separately, whereas other studies formally tested for an association. Out of the fourteen studies, four studies found a negative relationship between structural and functional brain abnormalities in depression *versus* control, two found a positive relationship, two found a mixed relationships, and six studies did not observe any relationship at all. The main results of these studies will be discussed and grouped by outcome. In addition, results have been described in relation to the neurobiological models for depression explained earlier.

### Negative Relationship

The oldest study investigated the influence of white matter abnormalities on functional activity in late-life depression (29). White matter burden was hypothesized to reduce structural connectivity between brain regions and might thereby interfere with information flow. The results showed no overall group differences between elderly depressed patients and non-depressed participants in global white matter abnormalities. Nevertheless, the fMRI-analysis identified increased responsivity to emotional faces in the subgenual ACC in depressed patients. Subsequently, a median split was used to distinguish between “low” and “high” amounts of white matter abnormalities between subjects in both groups. Patients with more white matter abnormalities showed more activity in the subgenual ACC compared to patients with little white matter abnormalities, whereas no differences were observed in elderly controls. Therefore, this study suggested that diffuse reductions in white matter integrity were associated with increased activity in the subgenual ACC. Moreover, because this brain region is part of the DMN, these results suggested a negative relationship: less widespread white matter integrity was associated with increased neural activity in the DMN.

A more direct correlation between white matter integrity and brain function was presented in another study (30); by using diffusion tensor imaging, authors found that integrity of the uncinate fasciculus, that connects the PFC with the medial temporal lobe, was reduced in depressed patients. Moreover, functional connectivity analysis obtained during the presentation of emotional faces showed increased connectivity between the subgenual ACC and medial temporal lobe structures (the amygdala and hippocampus). A correlation analysis found a larger reduction of uncinate fasciculus integrity was associated with a stronger increase in subgenual ACC-hippocampus connectivity in depressed patients, but not in healthy controls. As both the subgenual PFC and the medial temporal lobe are part of the DMN, this study also defined a negative relationship, whereby reduced localized structural integrity was associated with increased functional connectivity within the DMN.

Another study included in this review also focused on the subgenual ACC by investigating its volume and activity during an “N-back” working memory task (31) in depressed patients compared to controls. This task was expected to increase neural activity in the frontoparietal network and to reduce activity in the subgenual ACC. VBM-analysis in depressed patients showed volume reductions in the subgenual ACC and in the surrounding brain areas, such as the orbitofrontal cortex bilaterally, the temporal poles, and the left hippocampus and parahippocampal gyrus compared to controls. FMRI revealed task-related hypoactivation in the left lateral PFC and other regions, and failure of deactivation (meaning a relative increase in activity) in a subcallosal medial frontal cortical area in depressed patients, which area is part of the DMN and that included the subgenual ACC. Both clusters were in close proximity, but the cluster of failure of deactivation was more anterior in the medial frontal cortex compared to the region of volume reduction. Thus, although this study demonstrated no direct overlap between differences in structure and function, it did suggest a negative relationship: reduced subgenual ACC volume was associated with increased functional activity in (part of) the DMN.

Another study included in this review investigated the relation between cortical gyrification and task-based functional connectivity in patients with a recovered state of depression (32). There was significant bilateral hypogyrification in the depression group compared to healthy controls, extending across bilateral medial surface regions incorporating the precuneus. Additionally, there was hypergyrification in a more anterior region, incorporating the left dorsal ACC (dACC). A connectivity analysis of data acquired during a “go/no-go task” with the precuneus as seed region showed hyperconnectivity with a cluster in the right dorsomedial PFC and right frontal pole. A functional connectivity analysis with the dACC as seed region showed hypoconnectivity with a cluster in the left posterior temperoparietal cortex. Thus, connectivity from the precuneus (part of the DMN) that showed hypogyrification revealed hyperconnectivity, whereas connectivity from the dACC (part of the SN) that showed hypergyrification revealed hypoconnectivity. These findings suggest that differences in gyrification in patients who have recovered from depression are accompanied with functional connectivity differences in the opposite direction, thus again indicating a negative relationship: less localized structural brain volume (precuneus hypogyrification) is associated with increased functional connectivity in the DMN.

### Positive Relationship

A study that aimed to provide a more direct link between brain structure and functional connectivity correlated cortical thickness with functional connectivity across depressed patients compared with healthy controls (33). Initial unimodal group comparisons showed reduced cortical thickness in the dorsomedial PFC, dorsolateral PFC, rostral superior temporal gyrus, and posterior middle cingulate cortex. Reduced functional connectivity between the dorsomedial PFC and the precuneus and temporal cortex was found in depressed patients, as well as increased dorsomedial PFC connectivity with the left anterior insula and left middle frontal gyrus. Follow-up correlation analyses, combining cortical thickness and functional connectivity data, revealed that reduced dorsomedial PFC thickness was associated with reduced dorsomedial PFC connectivity with the precuneus in depression. These findings suggest a positive relationship: reduced dorsomedial PFC structure (part of the SN) is correlated with decreased functional connectivity in the precuneus (part of the DMN).

Another study used tensor-based morphometry as an index for local gray matter volume and intrinsic connectivity distribution as an index for the connectivity strength of a region with the rest of the brain (34). Unimodal results demonstrated reduced gray matter volume in the dACC and lower intrinsic connectivity in the ventromedial PFC, which did not overlap. In order to test for group differences in the correlation between brain structure and brain function across the entire prefrontal cortex, a single prefrontal structure-function correlation measure per subject was calculated. Again, the correlation between prefrontal brain volume and connectivity was positive in depressed patients (reduced volume correlated to reduced functional connectivity) and significantly stronger than in healthy controls.

### Mixed Relationship

One study combined structural MRI with RS-fMRI in patients with first-episode depression and treatment-resistant depression (35). The VBM-analyses showed reduced volume of the right middle temporal cortex in both patient groups and reduced bilateral caudate nucleus volume in the treatment-resistant group only. For first-episode patients, functional connectivity between the right middle temporal cortex and the left supramarginal gyrus was increased. Decreased functional connectivity was seen between the right middle temporal cortex and other areas: the right angular gyrus, left precuneus, and parahippocampal gyrus. For treatment-resistant patients, functional connectivity of the right middle temporal cortex was increased with the right precuneus, middle temporal gyrus, bilateral superior frontal gyri, and left middle frontal gyrus, and decreased with the right cuneus. In addition, the treatment-resistant group showed decreased connectivity of the caudate nucleus with the right middle orbitofrontal cortex and left occipital gyrus. Thus, this study showed mixed relationships, with both increased and decreased functional connectivity from brain regions with reduced structural brain volumes.

Standard correlational functional connectivity analyses cannot assess the direction of connectivity. Therefore, one study used Granger causality analysis to investigate the direction of the information flow during RS-fMRI (36). The authors had previously identified volume reductions in the left angular gyrus and the right inferior temporal gyrus using VBM (36). These areas were used as seed regions for the Granger causality analysis to investigate the influence of these seeds on other brain regions. In this analysis, Granger effects on brain regions that were positively correlated with the seed were considered “excitatory,” whereas Granger effects on brain regions that were negatively correlated with the seed were considered “inhibitory.” In comparison to healthy controls, the depressed patients showed an inhibitory effect from the left angular gyrus on the left superior temporal gyrus and the left inferior frontal gyrus. In addition, the right inferior temporal gyrus showed an inhibitory effect on the cerebellum and an excitatory effect on the right insula. These results suggest therefore both excitatory and inhibitory effects from brain regions with reduced volume, indicating mixed relationships between brain volume and functional connectivity.

### No Relationship

Five studies, that used VBM in combination with different functional methods, did not observe a significant relationship between structure and function abnormalities in the same brain regions. The results from the VBM-analysis of one study showed reduced gray matter volume in the right inferior temporal gyrus and left angular gyrus in depressed patients compared to healthy controls (37). In addition, the amplitude of low-frequency fluctuations (ALFF) was calculated from fMRI data which indexes the strength of regional resting-state connectivity. The results showed decreased low-frequency fluctuations in the left middle temporal gyrus, right superior temporal gyrus, and culmen. These locations did not overlap with the reductions in brain volume. This conclusion was supported by another study (38), however whereas Guo et al. (37) calculated ALFF as measure of local connectivity strength, Yang et al. (38) calculated regional homogeneity which indexes connectivity strength of neighboring voxels. The VBM-analysis showed increased gray matter volume in the posterior cingulate gyrus and decreased gray matter volume in the lingual gyrus in the depressed group. The functional analysis showed that regional homogeneity was increased in the precuneus and decreased in the putamen. Again, there was no overlap between the brain regions with differences in brain structure and functional connectivity.

Another study combined VBM with continuous arterial spin labeling (ASL) to assess regional cerebral blood flow (rCBF) (39). In depression, VBM-analysis showed reduced frontotemporal gray matter volume as well as reduced rCBF in the ACC and parahippocampal cortices, and increased rCBF in frontoparietal and striatal regions. These differences in rCBF remained after statistical correction for regional differences in brain volume, suggesting that altered neural activity at rest in depressed patients cannot be explained by structural differences.

Zhou et al. (40) combined VBM with resting-state global functional connectivity density as index of connectivity strength of a region with the rest of the brain. Depressed patients displayed reduced gray matter volume in different structures of the limbic system. In contrast, decreased functional connectivity density was mainly observed in the sensory system. Again, this study did not demonstrate any overlap between structural and functional abnormalities in depressed patients.

The VBM studies mentioned above relied on assessing the overlap of results that were obtained from independent analysis of structural and functional data. In contrast, He et al. (41) used a data-driven fusion method to directly combine VBM with correlations between resting-state networks, already during the analysis, in which the resting-state networks were identified with ICA. Although the results identified overlapping structural and functional brain differences in patients with bipolar disorder, the results only showed gray matter volume reductions in patients with unipolar depression in the cerebellum, amygdala, and hippocampus and no overlap with functional differences.

In addition to the studies that used VBM, Hermesdorf et al. (25) assessed white matter integrity of the corpus callosum using diffusion weighted images. This was combined with voxel-mirrored homotopic connectivity (VMHC) to assess functional connectivity between the same brain regions across hemispheres, which were anatomically connected through the corpus callosum. Patients with depression exhibited reduced VMHC in the cuneus, putamen, superior temporal gyrus, insula, and precuneus. Across patients and healthy controls, positive correlations were observed between callosal fractional anisotropy and connectivity in several of these clusters. Depressed patients and healthy controls did not however differ with regard to callosal fractional anisotropy, suggesting that there appeared no direct relationship between structural and functional connectivity.

### Heterogeneity of Included Studies

The grouping of studies used in our analysis (into the observed relationships: negative, positive, mixed, none) seems to suggest that more recent studies, compared studies published earlier, are more likely to report associations. We therefore performed a *post-hoc* comparisons using several study characteristics between studies that did find a significant relationship (negative, positive, or mixed) *versus* those that did. These comparisons showed that studies reporting some kind of relationship between structural and functional abnormalities tended to be older (year of publication; U = 9.5, P = 0.057), used smaller sample sizes (total N; U = 6.5, P = 0.024), used task-based fMRI more often [χ^2^(1) = 4.2, P = 0.040], tested a region-specific, model-driven hypothesis more often [χ^2^(1) = 4.7, P = 0.031], and tended to use a model-driven region-of-interest analysis more often [χ^2^(1) = 2.9, P = 0.086]. The origin of the study, however, showed no effect [Asian *vs.* other continents; χ^2^(1) = 2.4, P = 0.119].

## Discussion

Despite the extensive neuroimaging work that has been done over the past two decades, this systematic literature review revealed only fourteen studies trying to describe a significant relationship between brain structure and functional abnormalities in depressed patients *versus* controls. The relationships were heterogeneous or absent, whereby further quantitative analysis was limited due to the heterogeneity of studies. Surprisingly, a negative relationship between structural and functional brain abnormalities within the DMN was found, suggesting reduced gray or white matter integrity in association with enhanced neural activity or connectivity. It is remarkable that despite the fact that neuroimaging provides a promising method for (early) detection and exploration of the heterogeneous nature of depression, there is such a small number multimodal neuroimaging studies available examining depression. The use of different methodological approaches might account for the variability of the results presented in this systematic review.

### Included Neuroimaging Methods

The included studies showed reasonable consistency in the methods used to assess brain structure, but little consistency in the methods to assess brain function. Even though different structural imaging modalities were described (T1-weighted, T2-weighted, and diffusion weighted imaging), the majority of studies (64%) assessed regional gray matter volume using some form of VBM. Although a high percentage of studies (93%) used a form of fMRI to analyze brain function, there was little to no consistency in the use of specific analyses (RS-fMRI, task-based fMRI, seed-based, ICA-based, ASL, ALFF, VMHC). Therefore, the different relationships found in our systematic review (positive, negative, mixed, and none) could merely be due to variability in which brain structures and brain functions were examined. Interestingly, the studies that reported some link between abnormalities in brain structure and function in depressed patients, whether it was negative, positive, or mixed relationship, tended to be published earlier, used smaller samples, and tested more often region-specific hypotheses. In our opinion, this finding reflects the progress in the field of research in which the older studies could only test theory-based hypotheses, whereas more recent studies have used larger samples with sufficient power for hypothesis-free testing with stringent multiple comparisons correction. Nevertheless, our findings also suggest that potential relationships between brain structure and function might be specific to brain regions or certain aspects of brain function.

### Negative Relationship in Hypothesis-Based Studies

Four studies reported a negative relationship between brain structure and function; these studies used different measures of gray or white matter integrity and neural activity (or functional connectivity), but all focused on brain regions that were part of the DMN (29–32). These results were, however, not supported by five of the six other studies that reported null findings and included the DMN as well (37–41). This difference could be explained by the differing methodology used across the studies. Different studies might have tested other aspects of brain structure and function. Alternatively, because the studies that found a relationship between structure and function included smaller sample sizes and tended to use region-of-interest analyses more often, plausible statistical differences were present. The studies also differed in statistical power. Power was higher in hypothesis-driven region-of-interest analyses due to less stringent correction for multiple comparisons. This was also the case in the detection of false-positives, which can be seen in the fact that studies with smaller sample sizes had higher odds to produce false-positive results. Whether the differences in results were due to differences in neuroimaging methods or the statistical power can only be addressed in future studies that should aim to replicate the initial results using the same methods with appropriate sample sizes.

### Negative Relationship in Data-Driven Studies

The two studies that showed mixed relationships were data-driven, rather than based on the neurobiological models of depression (35, 37), making comparison with studies that targeted the DMN difficult. Nevertheless, the results could be considered in line with brain network specificity. This also applies to one study that found a positive relationship between structure and function focused on another network [i.e., the SN (33)]. The results however, from the study that reported a positive relationship (i.e., less prefrontal brain volume associated with reduced connectivity), were not consistent with this notion, as the DMN is also part of the prefrontal cortex (34). Probably, because the DMN only forms a small part of the entire prefrontal cortex, its contribution to those results was minimal.

### Clinical Implications

The negative relationship between brain structure and function, as reported in four of the multimodal studies, seems to be consistent with the unimodal studies. These studies often also reported reductions in structural integrity and increased functional connectivity (18, 42). Paradoxically, the negative relationship between structure and function was opposite to the positive relationship that was typically observed in healthy controls (2, 21, 22). But as hypothesis-driven studies in depressed patients aim to detect maximal differences with healthy controls, it is most likely that similar positive relations are also present in depressed patients. This suggested discrepancy may highlight a clinically important difference between brains of depressed patients *versus* healthy individuals. Replication of these negative structure-function-relationships in larger sample-sized studies using multimodal, data-driven analyses may lead to new, clinically helpful neuroimaging applications. These applications may facilitate prevention strategies in high-risk populations and improve (much earlier) detection of depressed (pre-)condition in patients, leading to earlier treatment with better outcomes. Also, more neuroimaging-based distinction between phenotypes of depression in patients may help to better understand the underlying pathophysiology. This in turn may also help to predict treatment outcome.

## Limitations of the Systematic Review

The limited number of studies that were currently available for inclusion prevented the possibility to draw firm conclusions and made it difficult to explain the variability in the results. Nevertheless, there are a couple of possible explanations for the differences in our findings. Firstly, as already discussed above, the brain regions and networks that were targeted differed across the various studies. Secondly, there were considerable differences in the clinical characteristics of the included patient groups (e.g., depressed *versus* recovered, adult *versus* elderly). Furthermore, different methods were used in the included studies to confirm the diagnosis MDD, which might have enhanced heterogeneity. Moreover, six out of fourteen studies included UD as well as BD patients. These patient groups should be considered to have had some similar (43) but also some different characteristics (44–46). Large heterogeneity in depressed patients in general, as was earlier mentioned in many unimodal studies (42), could explain the large differences in our findings (47). Investigation for smaller and more reliable units, such as individual symptoms (“in- or hypersomnia,” “agitation,” or “retardation”) might clarify the neuroimaging findings and its implications (48); e.g., the STAR*D study revealed 1,030 unique symptom profiles in 3,703 depressed patients (49). This could translate into many “neuroimaging profiles” that might by examined. Thirdly, the studies were cross-sectional and could not provide information about the cause or consequence of the observations. One study suggested that the negative relationship between brain structure and function persisted during recovery (32), which might indicate that this relationship was not directly related to the affective state but more likely a trait characteristic. It remained unclear whether the structural deficits had led to altered brain function, or whether differences in function had led to changes in brain structure. Finally, there was a large variation in the types of MRI-scans that were collected [e.g., structural MRI, diffusion tensor imaging (DTI), task-based fMRI, resting-state fMRI] and the types of MRI-data-analyses that were conducted (e.g., volumetry *vs.* gyrification, voxel-based analysis *vs.* tractography, activity *vs.* connectivity). Due to this large variability, it was not yet possible to pool the data and perform a quantitative meta-analysis.

## Conclusion

Although this systematic literature review did not find a strong relationship between structural and functional brain abnormalities in depressed patients *versus* controls, remarkably four studies reported negative relationships suggesting reduced gray or white matter integrity in association with enhanced neural activity or connectivity within the default mode network. This finding should, however, be considered preliminary because it needs to be tested in future large-scale, multimodal, data-driven neuroimaging studies. While neuroimaging techniques seem promising for the (early) detection and/or exploration of the heterogeneous nature of depression, it is surprising that to date so little multimodal neuroimaging studies have been conducted in depressed patients. Hopefully in the future modern multivariate techniques may help in the analysis of these multimodal data sets in order to help establish generalizable predictions about patients.

## Author Contributions

DS and GV performed a systematic search. DS, GW and JW wrote this systematic review and all authors helped to draft the manuscript. All authors read and approved the final manuscript.

## Funding

This study was supported by the Dutch Brain Foundation (Nederlandse Hersenstichting).

## Conflict of Interest

The authors declare that the research was conducted in the absence of any commercial or financial relationships that could be construed as a potential conflict of interest.
