# Supplementary material for: The Link Between Structural and Functional Brain Abnormalities in Depression: A Systematic Review of Multimodal Neuroimaging Studies
Source: Front Psychiatry. 2020 Jun 3;11:485. doi: 10.3389/fpsyt.2020.00485 (PMC7283615; doi:10.3389/fpsyt.2020.00485)
Supplement: Supplementary file 1 [file DataSheet_1.docx]

# Supplementary Tables

# **Table 1:** Medline search terms

| # | Searches | Results |
| --- | --- | --- |
| 1 | depressive disorder, major/ | 28169 |
| 2 | (mdd or Major Depressive or major depression or unipolar or bipolar or ((depression or depressive) adj3 disorder?)).ab,kf,ti. | 125096 |
| 3 | 1 or 2 [MDD] | 133077 |
| 4 | exp magnetic resonance spectroscopy/ | 212417 |
| 5 | (magnetic resonance spectroscopy or nmr spectroscopy or mr spectroscopy or magnetic resonance spectrometry or nmr spectrometry or mr spectrometry).ab,kf,ti. | 66222 |
| 6 | (arterial spin or asl or bold or blood oxygen dependent).ab,kf,ti. | 15607 |
| 7 | (functional connectivity or functional mri or structural mri or (functional adj3 (neuroimaging or imaging))).ab,kf,ti. | 63235 |
| 8 | (function* adj10 (brain or cortex or cortical or lobe? or hippocampus or amygdala or limbic)).ab,kf, ti. | 134326 |
| 9 | or/4-8 [functional brain abnormalities] | 414030 |
| 10 | (structure or structural).ab,kf,ti. | 1493545 |
| 11 | diffusion tensor imaging/ | 7629 |
| 12 | (dti or diffusion tensor).ab,kf,ti. | 16431 |
| 13 | or/10-12 [structural brain abnormalities] | 1506052 |
| 14 | and/3,9,13 | 1071 |
| 15 | remove duplicates from 14 | 944 |

# **Table 2:** Embase search terms

| # | Searches | Results |
| --- | --- | --- |
| 1 | major depression/ | 53232 |
| 2 | (mdd or Major Depressive or major depression or unipolar or bipolar or ((depression or depressive) adj3 disorder?)).ab,kw,ti. | 163405 |
| 3 | 1 or 2 [MDD] | 177274 |
| 4 | nuclear magnetic resonance spectroscopy/ | 111524 |
| 5 | (magnetic resonance spectroscopy or nmr spectroscopy or mr spectroscopy or magnetic resonance spectrometry or nmr spectrometry or mr spectrometry).ab,kw,ti. | 72641 |
| 6 | arterial spin labeling/ | 1121 |
| 7 | (arterial spin or asl or bold or blood oxygen dependent).ab,kw,ti. | 21515 |
| 8 | (functional connectivity or functional mri or structural mri or (functional adj3 (neuroimaging or imaging))).ab,kw,ti. | 78394 |
| 9 | functional magnetic resonance imaging/ | 63620 |
| 10 | (function* adj10 (brain or cortex or cortical or lobe? or hippocampus or amygdala or limbic)).ab,kw,ti. | 166792 |
| 11 | or/4-10 [functional brain abnormalities] | 389544 |
| 12 | (structure or structural).ab,kw,ti. | 1552016 |
| 13 | diffusion tensor imaging/ | 21140 |
| 14 | (dti or diffusion tensor).ab,kw,ti. | 22862 |
| 15 | or/12-14 [structural brain abnormalities] | 1570910 |
| 16 | and/3,11,15 | 1871 |
| 17 | remove duplicates from 16 | 1771 |

# **Table 3**: PsycINFO search terms

| # | Searches | Results |
| --- | --- | --- |
| 1 | major depression/ or reactive depression/ or recurrent depression/ or treatment resistant depression/ or bipolar disorder/ | 129516 |
| 2 | (mdd or Major Depressive or major depression or unipolar or bipolar or ((depression or depressive) adj3 disorder?)).ab,id,ti. | 95164 |
| 3 | 1 or 2 [MDD] | 159207 |
| 4 | exp magnetic resonance imaging/ | 36771 |
| 5 | (magnetic resonance spectroscopy or nmr spectroscopy or mr spectroscopy or magnetic resonance spectrometry or nmr spectrometry or mr spectrometry or dti or diffusion tensor or arterial spin or asl or bold or (blood oxygen adj2 dependent) or functional connectivity or functional mri or structural mri or fmri or (functional adj3 (neuroimaging or imaging)) or (function* adj10 (brain or cortex or cortical or lobe? or hippocampus or amygdala or limbic))).ab,id,ti. | 101527 |
| 6 | or/4-5 [functional brain abnormalities] | 111635 |
| 7 | (structure or structural).ab,id,ti. [structural brain abnormalities] | 250845 |
| 8 | and/3,6-7 | 1198 |

# **Table 4.** Neuroimaging characteristics of the included studies.

| Author/Publisher | Structural MRI | Functional MRI | Task-based | Functional Connectivity | ROI/Region ypothesis |
| --- | --- | --- | --- | --- | --- |
| Aizenstein et al 2011 *American journal of Psychiatry* | WMHV | fMRI, emotional faces | + | - | +/+ |
| Ma et al, 2012  *Plos ONE* | VBM | fMRI, SBC | - | + | -/- |
| De Kwaasteniet et al, 2013  *Biological Psychiatry* | DTI, FA | fMRI, SBC | + | + | +/+ |
| Van Tol et al, 2013  *Psychological Medicine* | cortical thickness | fMRI, fALFF | - | + | +/+ |
| Guo et al, 2014  *Prog Neuropsycholopharamcol Biol Psychiatry* | VBM | fMRI, fALFF | - | + | -/- |
| Rodriquez-Cano et al, 2014  *Psycholgical Medicine* | VBM | fMRI, N-back task | + | - | -/+ |
| Guo et al, 2015  *Journal of Affective Disorders* | VBM | fMRI, ICA | - | + | -/- |
| Hermesdorf et al, 2015  *Human Brain Mapping* | DTI, FA | fMRI, VMHC | - | + | -/- |
| Nixon et al, 2015  *British Journal of Psychiatry* | LGI’s | fMRI, SBC | + | + | +/+ |
| Vasic et al, 2015  *Journal of Psychiatry and Neuroscience* | VBM | Perfusion CASL, regional-CBF | - | - | +/+ |
| Yang et al, 2015  *Neuropsychiatric Disease and Treatment* | VBM | fMRI, ReHo | - | + | -/- |
| He et al, 2017  *Brain structure and function* | VBM | fMRI, joint-ICA | - | + | -/- |
| Scheinost et al, 2018  *Neuropsychopharmacology* | TBM | fMRI, intrinsic connecitivity distribution | - | + | +/+ |
|  |  |  |  |  |  |
| Zhuo et al, 2017  *Brain Imaging and Behavior* | VBM | fMRI, rs-global functional connectivity density | - | + | -/- |

Task-based=whether the imaging data were obtained during performance of a task or during rest; functional connectivity=whether the primary analysis was conducted on connectivity analysis or not; ROI/Region hypothesis=whether an ROI analysis was performed/whether a region- or network-specific hypothesis was tested.

***Abbreviations***

CASL= continuous arterial spin labeling, CBF= cerebral blood flow, DTI=diffusion tensor imaging, FA=fractional anisotropy, fALFF= fractional amplitude of low-frequency fluctuation, ICA= independent component analysis, LGI=Local gyrification index, ReHo=regional homogeneity, SBC=seed-based correlation, TBM=tensor-based morphometry, VBM=voxel-based morphometry, VMHC=voxel-mirrored homotopic connectivity, WMHV= white matter hyperintensity volume

# **Table 5**. Clinical characteristics and main results from the included studies.

| Author/Publisher | Continent | Participants | Age (mean or range) | Polarity | Localisation | Relation |
| --- | --- | --- | --- | --- | --- | --- |
| Aizenstein et al 2011 *American journal of Psychiatry* | North-America | 33 depression.  27 Controls | 71.6 depression  67.7 controls | unipolar | subgenual ACC (DMN) | Negative |
| Ma et al, 2012  *Plos ONE* | Asia | 18 depression treatment resistent  17 depression Therapy-responsive  17 Controls | 18-50 | unipolar | Right middle temporal cortex, bilateral caudate nucleus, right middle temporal cortex, supramarginal gyrus, right angular gyrus, left and right precuneus, parahippocampal gyrus, middle temporal gyrus, bilateral superior frontal gyri, left middle frontal gyrus, right cuneus, caudate nucleus, right middle orbitofrontal cortex and left occipital gyrus | Mixed |
| De Kwaasteniet et al, 2013  *Biological Psychiatry* | Europe | 18 depression  24 Controls | 44.6 depression.  40.2 controls | Unipolar/bipolar | ucinate fasciculus/subgenual ACC, hippocampus, amygdala (DMN) | Negative |
| Van Tol et al, 2013  *Psychological Medicine* | Europe | 20 depression  20 Controls | 38.25 depression  33.75 controls | unipolar | Precuneus (DMN)  dorsomedial PFC (SN) | Positive |
| Guo et al, 2014  *Prog Neuropsychopharmacol Biol Psychiatry* | Asia | 44 depression.  44 Controls | 27.5 depression  29.4 controls | unipolar |  | None |
| Rodriquez-Cano et al, 2014  *Psycholgical Medicine* | Europe | sMRI: 32 depression /64 controls  fMRI: 26 depression /52 Controls | 18-70 | unipolar | subgenual ACC, orbitofrontal cortex, left hippocampus and parahippocampal gyrus, left lateral PFC (DMN) | Negative |
| Guo et al, 2015  *Journal of Affective Disorders* | Asia | 44 depression  44 Controls | 27.5 depression  29.4 controls | unipolar | Left angual gyrus, right inferior temporal gyrus used as seeds | Mixed |
| Hermesdorf et al, 2015  *Human Brain Mapping* | Europe | 368 depression  461 Controls | 35-65 | unipolar |  | None |
| Nixon et al, 2015  *British Journal of Psychiatry* | Europe | 20 depression (recovered)  20 Controls | 24-63 | unipolar/bipolar | precuneus, left dorsal ACC, right dorsomedial PFC, right frontal pole (DMN)  dorsal ACC(SN) | Negative |
| Vasic et al, 2015  *Journal of Psychiatry and Neuroscience* | Europe | 43 depression  29 controls | 34.5 depression  37.1 controls | unipolar/bipolar |  | None |
| Yang et al, 2015  *Neuropsychiatric Disease and Treatment* | Asia | 50 depression  50 controls | 31.1 depression  31.3 controls | unipolar |  | None |
| He et al, 2017  *Brain structure and function* | North-America | 40 depression  33 Controls | 35.2 BD depression.  35.2 UD depression  33.7 controls | unipolar/bipolar |  | None |
| Scheinost et al, 2018  *Neuropsychopharmacology* | North-America | 17 depression  20 Controls | 34.4 depression  32.6 controls | unipolar/bipolar | entire prefrontal cortex (DMN**)** | Positive |
| Zhuo et al, 2017  *Brain Imaging and Behavior* | Asia | 45 depression  48 Controls | 38.8 depression  38.6 controls | unipolar/bipolar |  | None |

Localisation=the brain regions where an association or overlap between structural and functional brain abnormalities were observed; Relation=the direction of association between structural and functional abnormalities.
